# Physicochemical Characteristics of Biofuel Briquettes Made from Pecan (*Carya illinoensis*) Pericarp Wastes of Different Particle Sizes

**DOI:** 10.3390/molecules27031035

**Published:** 2022-02-03

**Authors:** Maginot Ngangyo Heya, Ana Leticia Romo Hernández, Rahim Foroughbakhch Pournavab, Luis Fernando Ibarra Pintor, Lourdes Díaz-Jiménez, Michel Stéphane Heya, Lidia Rosaura Salas Cruz, Artemio Carrillo Parra

**Affiliations:** 1 Agronomy School (FA), Universidad Autónoma de Nuevo León (UANL), Francisco Villa S/N, Col. Ex-Hacienda “El Canadá”, Escobedo 66050, Nuevo Leon, Mexico; nheyamaginot@yahoo.fr (M.N.H.); ana.romohrnd@uanl.edu.mx (A.L.R.H.); 2 Department of Botany, Biological Science School (FCB), Universidad Autónoma de Nuevo León (UANL), Ave. Pedro de Alba S/N Cruz & Ave. Manuel L. Barragán, San Nicolas de los Garza 66451, Nuevo Leon, Mexico; rahim.forough@gmail.com; 3 Facultad de Ingeniería en Tecnología de la Madera, Universidad Michoacana de San Nicolás de Hidalgo, Gral. Francisco J. Múgica S/N Ciudad Universitaria, Morelia 58040, Michoacan, Mexico; luis.pintor@umich.mx; 4 Laboratorio de Revaloración de Residuos, Sustentabilidad de los Recursos Naturales y Energía, Cinvestav Saltillo, Ramos Arizpe 25900, Coahuila, Mexico; lourdes.diaz@cinvestav.edu.mx; 5 Biotechnology Institute, Biological Science School (FCB), Universidad Autónoma de Nuevo León (UANL), Ave. Pedro de Alba S/N Cruz & Ave. Manuel L. Barragán, San Nicolas de los Garza 66451, Nuevo Leon, Mexico; 6 Institute of Silviculture and Wood Industry (ISIMA), Universidad Juárez del Estado de Durango (UJED), Boulevard del Guadiana 501, Ciudad Universitaria, Torre de Investigación, Durango 34120, Durango, Mexico

**Keywords:** solid waste-to-biofuel, biomass densification, granulometric distribution, proximate analysis, functional groups for energy storage

## Abstract

Pecan nut (*Carya illinoensis*) pericarp is usually considered as a waste, with no or low value applications. Its potential as a densified solid biofuel has been evaluated, searching for alternatives to generating quality renewable energy and reducing polluting emissions in the atmosphere, based on particle size, that is an important feedstock property. Therefore, agro-industrial residues from the pecan nut harvest were collected, milled and sieved to four different granulometry: 1.6 mm (N° 12), 0.84 mm (N° 20), 0.42 mm (N° 40), and 0.25 mm (N° 60), used as raw material for biofuel briquette production. The carbon and oxygen functional groups in the base material were investigated by Fourier transform infrared spectroscopy (FTIR) and proximate analyses were performed following international standards, for determining the moisture content, volatile materials, fixed carbon, ash content, and calorific value. For the biofuel briquettes made from base material of different particle sizes, the physical characteristics (density, hardness, swelling, and impact resistance index) and energy potential (calorific value) were determined to define their quality as a biofuel. The physical transformation of the pecan pericarp wastes into briquettes improved its quality as a solid biofuel, with calorific values from around 17.00 MJ/kg for the base material to around 18.00 MJ/kg for briquettes, regardless of particle size. Briquettes from sieve number 40 had the highest density (1.25 g/cm^3^). Briquettes from sieve number 60 (finest particles) presented the greater hardness (99.85). The greatest susceptibility to swelling (0.31) was registered for briquettes with the largest particle size (sieve number 20). The IRI was 200 for all treatments.

## 1. Introduction

Pecan (*Carya illinoensis*) nuts are dry fruits native to California in the US, with the biggest production coming from Mexico (around 124,000 tons/year) [1]. Pecan nut processing generates important amounts of waste in the form of shells and pericarp (40–50%) [2,3] that are generally dumped into the environment or burned, representing an important loss of biomass and, at the same time, constituting a cause of some environmental problems. For these wastes to be better used it is necessary to know their composition and some characteristics [4,5].

It is well known that agro-industrial wastes, as well as forest residues, municipal solid wastes, and refused-derived fuels represent different types of biomass energy resources [6]. Nevertheless, their direct combustion has negative aspects owing to their intrinsic properties, such as low density, low calorific value per unit volume, and high moisture content. Moreover, the direct burning of agricultural residues is inefficient due to associated transportation, storage, and handling problems [7,8]. Therefore, it is necessary to develop strategies for converting biomass to secondary fuels with better characteristics than the base material [9]. Currently, briquetting is one of the suitable evolving technologies of waste materials conversion to solid biofuels for energy purposes. Thus, biofuel briquettes provide a sustainable approach for the improvement and efficient utilization of agricultural and/or other biomass residues [10].

However, biomass briquetting depends on feed parameters that influence the extrusion process. The two most important feedstock properties are particle size and moisture content [11]. For moisture content, the optimal level is defined by the mandatory technical standard EN 18134-2, while for particle size, which is also considered as great influencer of final briquette quality [12], there is no mandatory technical standard to define optimal range of particle size. Furthermore, briquette hardness and quality can be checked by water test, where a quality briquette should fall to the bottom in a moment due to its higher specific density than water [13].

According to Jenkins et al. [14], these physical properties are closely linked to molecular structure, affecting the combustion characteristics of a particular fuel or fuel blend. In this way, the functional groups prevalent in biomass derived molecules may be related to the fuel properties such as combustion characteristics and emissions, being carbon and oxygen functional groups the bio-derived molecules with a real application as fuels [14]. The retention of oxygen atoms, abundant in biomass, provides certain advantages, including complete fuel combustion and less harmful exhaust emissions [15,16]. For carbon atoms that are the molecular building blocks of cellulosic biomass, the number is limited to 5- and 6-carbon containing species. The effect of chain extension must also be considered, especially for more specialized applications [14].

Pecan waste products are rich carbon sources [17], with high concentrations of tannins and phenols [18,19]. The lignocellulosic composition of these pecan wastes, in terms of their content in cellulose, hemicellulose, lignin, and ash from the shells, are 5.6, 3.8, 70, and 5.85%, respectively [17]. In the case of pericarp they are 30, 26, 41, and 1.7% [20], and from the pecan branches they are 38.7, 30.2, 23.3, and 0.4%, respectively [21].

Although pecan wastes have been studied as a feedstock for bioenergy purposes [22], and their main components have been determined [23,24], specific information on their properties for biofuel briquettes elaboration is scarce. Therefore, the present study aimed to determine the most appropriate particle size from pecan pericarp feedstock materials for biofuel briquettes production, to optimize the briquetting process, for the prevention of material lose during production, transportation, and storage; and to investigate how the energy content and fuel quality of pecan briquettes differ among the functional groups.

## 2. Results

### 2.1. Proximate Analysis of the Pecan Pericarp Base Materials

**Figure 1:**
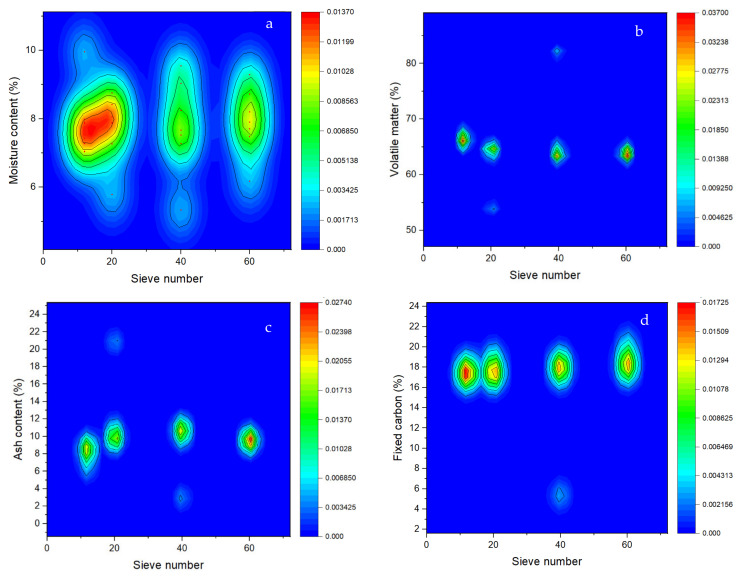
Results of the proximate analyses of pecan pericarp wastes of different particle sizes: 1.6 mm (N° 12), 0.84 mm (N° 20), 0.42 mm (N° 40) and 0.25 mm (N° 60). (**a**)—Moisture content, (**b**)—Volatile matter, (**c**)—Ash content, (**d**)—Fixed carbon.

The results of the proximate analyses of the base materials from pecan pericarp residues are presented in Figure 1. The moisture content of the four analyzed granulometry 1.6 mm (sieve N° 12), 0.84 mm (sieve N° 20), 0.42 mm (sieve N° 40), and 0.25 mm (sieve N° 60), did not present significant differences, as the probability (*p* value) were 0.993, with values ranging between 7.63 and 7.92% (Figure 1a), values that are <12% as indicated by the EN-14774-1 standard [25]. The highest moisture content was recorded in granulometry 1.6 mm (N° 12) and 0.84 mm (N° 20), corresponding to the finest materials.

Regarding the volatiles, there were no significant differences (*p* value = 0.595) among the granulometric numbers analyzed, with values ranged between 63.01 and 66.68% (Figure 1b), being below that established by the UNE-EN-15148 standard [26]. The finest material (particle size N° 12) presented the highest content of volatiles.

For the ash content, there were no significant differences between the treatments (*p* value = 0.156). The highest ash content was presented with granulometry 0.42 mm (N° 40), and the lowest ash content was presented in granulometry 1.6 mm (N° 12), with values of 11.55% and 8.20%, respectively (Figure 1c). These values are outside the UNE-EN-14775 standard [27].

The fixed carbon did not show significant differences between the granulometry (*p* value = 0.727). The fixed carbon content recorded the range from 16.09 to 18.59%, values that correspond to particle sizes N° 40 (0.42 mm) and N° 60 (0.25 mm), respectively (Figure 1d). The finest materials presented very similar values (17.49% and 17.73 for materials of particle size 12 and 20, respectively).

### 2.2. Energy Content of the Base Materials and the Resulting Briquettes, at Different Particle Sizes, from Pecan Pericarp Residues

**Figure 2:**
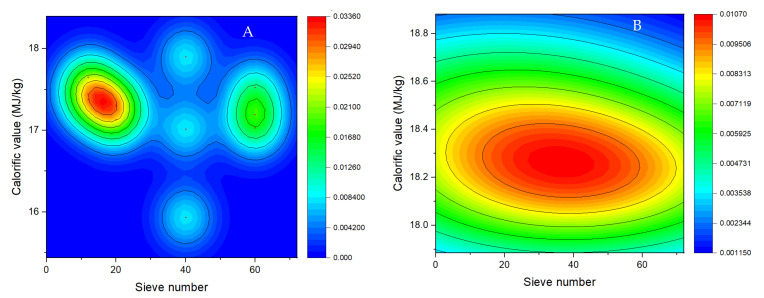
Calorific values of pecan pericarp residues at the different particle sizes 1.6 mm (N° 12), 0.84 mm (N° 20), 0.42 mm (N° 40) and 0.25 mm (N° 60). (**A**). Raw material; (**B**). Briquettes.

The calorific value analysis shows no significant differences in particle size between the base material (*p* value = 0.50) and the briquettes (*p* value = 0.66). The calorific value ranged from 17.04 to 17.54 MJ/kg for the base material (Figure 2A), and from 18.19 to 18.45 MJ/kg for the briquettes (Figure 2B), which suggests that the physical transformation of the pecan pericarp raw material into biofuel briquettes considerably improves its calorific potential. However, the highest calorific value was registered for the smallest particle sizes with the raw material (0.42 mm and 0.25 mm, corresponding to sieves number 40 and 60, respectively). At the same time, the briquettes presented a similar trend of calorific value for the materials from the different particle sizes analyzed, with greater energy power. This means that the size of particles is decisive for the energy value of a biofuel, since it affects the contact among particles, smaller particles has more contact surface and thus heat transmission is easy.

These calorific values are within the range established by the EN-14918 standard [28], both for the base material and for the resulting bio-briquettes. This means that the base material from pecan pericarp residues already constitutes an interesting source of biofuel, which would be more efficient if transformed into briquettes.

### 2.3. Morphological Characteristics of the Different Particle Sizes from the Pecan Pericarp Residues

**Figure 3:**
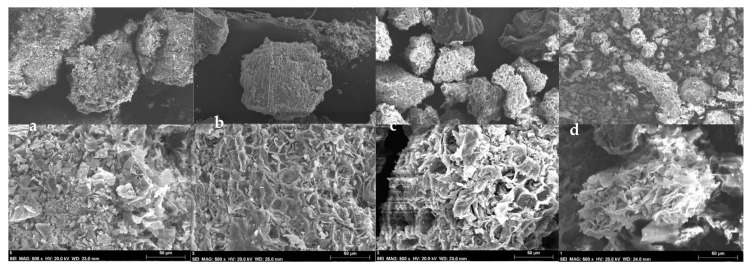
Typical morphology of the particle sizes in samples from pecan pericarp residues. (**a**)—Sieve number 12 (1.6 mm); (**b**)—N° 20 (0.84 mm); (**c**)—N° 40 (0.42 mm); (**d**)—N° 60 (0.25 mm).

Images from the scanning electron microscopy (SEM) analysis of pecan pericarp particle sizes are presented in Figure 3, showing that the different particles are ovoid in shape and of homogeneous size as a result of a correct reduction process. This homogeneity in the shapes and sizes of the particles is important, since there is greater cohesion at a homogeneous size.

### 2.4. Fourier Transform Infrared (FTIR) Analysis of Functional Groups in the Pecan Pericarp Residues

**Figure 4:**
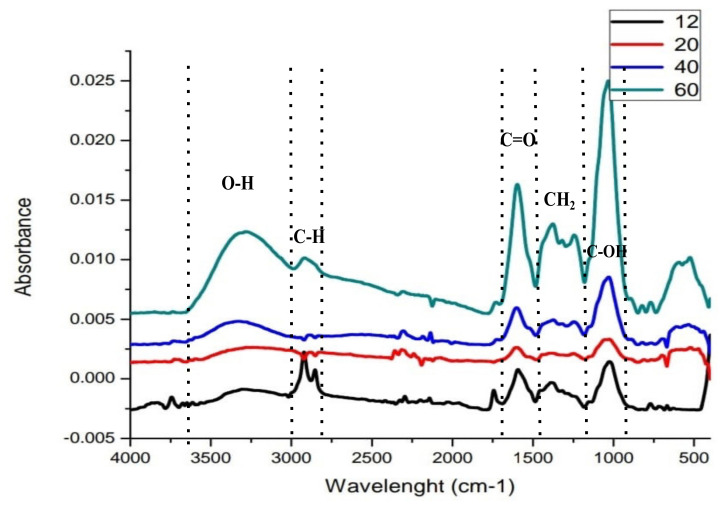
FT-IR spectrograms of different particle sizes from pecan pericarp residues, indicating the stretching of the signals corresponding to the most representative functional groups.

The main functional groups present in the pecan pericarp residues are shown in Figure 4, where the highest stretching can be observed in the particles size 0.25 mm (N° 60), and the lowest stretching, in the particle sizes 1.6 mm (N° 12). However, the trend of the signals by the functional groups is the same in the material from all the particle sizes, being the band between 3000 and 3600 cm^−1^ attributed to the stretching of hydroxyl (OH) groups, and the band registered between 2750 and 3000 cm^−1^, corresponding to methyl (CH_3_) groups. The peak at 1600 cm^−1^ corresponds to the carbonyl group (C=O), with the corresponding stretching vibration from 1490–1615 cm^−1^. The stretching signals at 1200–1490 cm^−1^ are attributed to the methylene groups (CH_2_), which show a peak at 1450 cm^−1^. A higher absorbance characterized the carboxyl group (COOH), reaching its peak at 1000 cm^−1^, within the stretching vibration from 700–1200 cm^−1^. However, as far as the CH functional group is concerned, the absorbance differs greatly between particles, there were two well-defined peaks in the particles of N° 12. At the same time, in 20 and 40 particle size, that peaks practically disappeared, and in particle size N° 60, it reappeared but one alone, in lower intensity.

**Figure 5:**
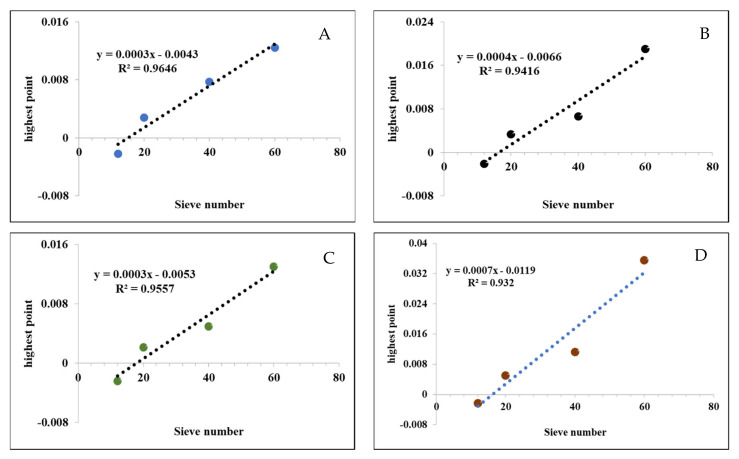
Numerical materialization of the intensity of the most representative signals of the FT-IR spectrograms as a function of particle size. (**A**) hydroxyl groups (O-H); (**B**) carbonyl group (C=O); (**C**) methylene group (CH_2_); (**D**) carboxyl group (C-OH).

The intensity of the infrared spectrogram bands was materialized numerically by obtaining the highest point through the OriginLab program. As Figure 5 shows, the intensity of the spectrometric stretching was particle size-dependent, being the highest values obtained with the particles size 60 and the lowest values with the particle size 12, for all the functional groups (O-H, C=O, CH_2_, C-OH).

### 2.5. Physical Properties of the Biofuel briquettes from Different Particle Sizes of Pecan Pericarp Residues

**Figure 6:**
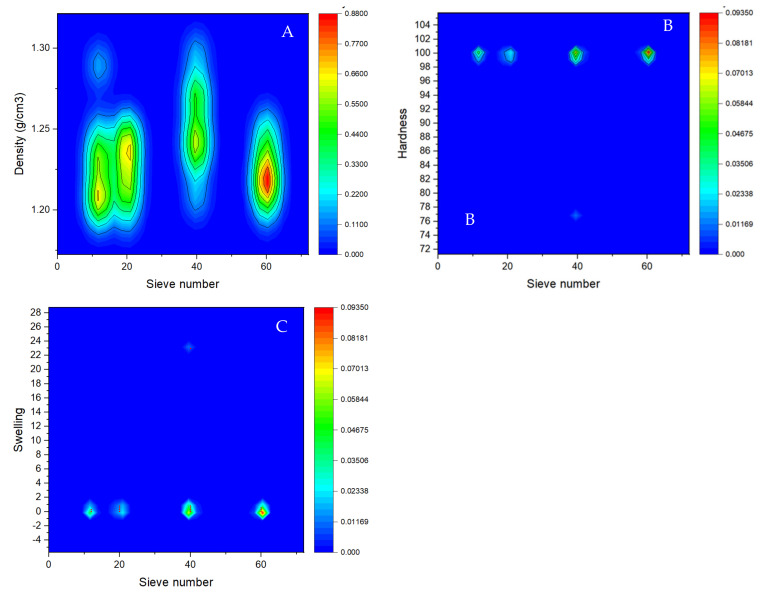
Physical characteristic of biofuel briquettes from pecan pericarp wastes of different particle sizes. (**A**) Density; (**B**) Hardness; (**C**) Swelling.

At the 0.05 level, the density means of biofuel briquettes from pecan pericarp are significantly different (*p* value = 0.02), with values from 1.22 to 1.25 g/cm^3^. Briquettes from sieve number 40 had the highest density, while sieve numbers 12 and 20 had the lowest density (Figure 6A), suggesting that thicker materials produce lower-density briquettes than finer materials. However, the appropriate particle size for briquettes of good density is 0.42 mm material from the sieve number 40, since with the smallest particle size 0.25 mm (sieve number 60, the density dropped to 1.23 g/cm^3^. This trend is the same with hardness, where the finest particles presented greater resistance to impact, with a hardness of 99.85 for biofuel briquettes from particle size of sieve number 60 (Figure 6B).

Regarding the swelling index, the briquettes with the largest particle size presented the greatest susceptibility to swelling, with a value of 0.31 for material from sieve number 20. In contrast, briquettes from sieve number 60 presented a value of 0.15 (Figure 6C).

After dropping briquettes three times from 1.85 m height, they did not lose weight so there were no broken pieces. Therefore, the IRI was 200 for all treatments, representing the maximum value that can be presented.

## 3. Discussion

### 3.1. Characteristics of the Pecan Pericarp Raw Material at Different Particle Sizes for Energy Use

The characterization of the base material from pecan pericarp residues was done through proximate analysis, determining the moisture, volatile, ash contents, and fixed carbon. The moisture was around 7.8%, meeting the requirements of the EN-14774-1 standard [25], which established the moisture content in the feed biomass as a very critical factor, and that may be around 10–12%. The moisture present in biomaterials forms steam under high-pressure conditions, which then hydrolyses the hemicellulose and lignin into lower molecular carbohydrates, lignin products, sugar polymers, and other derivatives [9], hence the need to keep the moisture content at a specific value. For five analyzed biomaterials (rapeseed oilcake, pine sawdust, Virginia mallow chips, Rape straw, and Willow chips), Stolarski et al. [29] reported a moisture content range from 9.8 to 18.3%. For palm oil residues (shell and fiber), Husain et al. [30] pointed out a moisture content of 12%. Chen et al. [31] suggested that the moisture content should be range between 10 and 15% for biomaterials of energy purposes. According to Kaur et al. [9], more than 15% moisture content produces poor and weak briquettes. Felfli et al. [32] advocate producing briquettes from agro-industrial materials (e.g., rice husk, coffee husk, bagasse, soybean husk, or sawdust) for their moisture content of 15%.

As for the volatile, the values oscillated around 65%, being below that established in the UNE-EN-15148 standard [26]. The volatile compounds come from both the organic part of the biomass and the inorganic part; and a high content of these compounds indicates the presence of an organic load when they are in a range of 74–89%, which provides a susceptibility to thermal degradation in processes such as combustion and pyrolysis. However, a low material volatile content is advantageous since it reduces gases emissions such as condensable or not condensable containing CO, CO_2_, CH_4_ during combustion. Barroso [33] found a lower percentage of volatile matter that volatilizes during combustion, concluding that a high percentage does not volatilize. This may justify the percentage obtained in this study, lower than what is established by the standard.

The percentage of volatiles that were not released during combustion can explain the high value obtained for the ashes of the pecan pericarp, which were 8–12%, above the maximum permissible limits of international standards. However, fuels with levels of more than 20% ash are not enabling for heat generation due to the residuality of chemical compounds that interfere in the combustion process.

Low fixed carbon content was found in the pecan pericarp (16.09–18.59%). According to Demirbas [34] and Ngangyo-Heya [35], the low content of fixed carbon increases friability and brittleness, decreasing compressive strength, cohesion, and energy.

However, the calorific value of the walnut pericarp (around 17.00 MJ/kg) resulted acceptable for the production of the second-generation solid biofuel, thus constituting an alternative to protect forests from clearing for the production of charcoal.

Although not all the properties were fully met for the generation of quality biofuels, the ash content of pecan pericarp was slightly high. On the other side, the fixed carbon and the volatile matter were little lower than the normal. The values obtained, especially moisture content and the calorific value, meet the standard ranges established by the standardized norms, constituting pecan pericarp materials as suitable for their use as biofuels. However, the improvement of its characteristics by densification may allow the development of eco-friendly materials, since they are renewable and abundant resources, according to Shekar and Ramachandra [36] and Spiridon et al. [37].

FTIR is one of the most used techniques for characterization of lignocellulosic materials, since it is able to identify the main functional groups that are present in its constitution, to analyze the relationship of some bands through lateral order index and total crystalline index, which allow understanding the crystalline cellulose structure and the influence of crystalline and amorphous domains on the properties of those materials. It is worth mentioning that in the last decade, this method has been widely used for the identification and quantification of compounds in solid mixtures (e.g., soil, plant extracts, etc.) as well as liquids. The strong absorption registered in the materials of particle size 60 suggests a high bioavailability of the functional groups in the finer lignocellulosic materials than in the thick ones. Biomass produces pure linear hydrocarbons, where hexane and pentane were reported to be the main components of cellulose [38]. It is reported that an increase in the number of carbon atoms in a fuel molecule has a profound effect on a range of physical properties, owing to the increase in intermolecular forces related to the surface area of the molecule. This could be seen in particle size change of the pecan pericarp which, although it was a physical modification of the material, presented a greater amount of the functional groups OH, CH, C=O, C-OH in the smaller particles (sieve N° 60), indicating the effect of the mill (raising the temperature), which could have caused the rupture of certain structural elements that facilitated the presence of greater bonds as mentioned. The carbon functional groups are reported to have a profound effect on melting, flash, and boiling points while keeping the carbon number the same. As intermolecular forces increase with increasing carbon number, the density, boiling point, melting point, and flash point.

### 3.2. Physical and Energy Properties of Biofuel Briquettes from Pecan Pericarp Residues at Different Particle Sizes

The briquettes for energy use correspond to a solid fuel of renewable origin, obtained by the densification of biomass. They are compressed at high pressure without the presence of additives, obtaining a homogeneous product with very low humidity [33]. The main characteristic of briquettes is their high density and homogeneity [33]. Density is an important parameter to characterize the briquetting process. The standards define the interval of briquette density values from 1 to 1.4 g/cm^3^ [39], which means that the materials of all particle sizes used in this study are suitable to produce good quality briquettes. However, it was found that the briquettes made from materials with larger particle sizes presented the lowest density, compared to briquettes produced with fine materials, due to the spaces left by larger particles, while in briquettes with smaller particles, the spaces are reduced. This agrees with the results of Tirado [40], who found that the compaction process, which consists of the application of pressure and temperature, causes a decrease in the volume of the walnut shell particles, increasing the density of the briquettes. The suitable material, as the present study suggests, was particles of size N° 40 (0.42 mm) to reach the highest density. Higher density leads to a higher energy/volume ratio desirable in terms of transportation, storage, and handling. Briquettes with higher density have a longer burning time [9]. The density of bio-waste briquettes depends on the density of the original bio-waste, the briquetting pressure, and, to a certain extent, on the briquetting temperature and time.

Regarding the homogeneity, Tirado [40] found that the smaller the particle size, the briquettes have greater homogeneity, with which he established an optimal particle size of 2 mm. This further shows that for the densification process, the particle sizes are of great importance, since it has been shown that as the particle size increases, there is a decrease in the bonding force between particles, and the speed of heat transmission is reduced due to the void spaces. Jha and Yadav [41] proposed a mixture of particle sizes. They found that different sizes of particles improve the packing dynamics and contribute to high static strength.

The hardness and impact resistance are other characteristics that determine the physical quality of briquettes, since they simulate the compression force due to the weight of the other briquettes that can be supported during storage or transport. According to previous studies, finer grinding of feedstock material results in solid biofuels of higher quality [42,43]. MacBain [44] asserted that larger particles accept less moisture which causes fractures in solid biofuels in contrast with finer particles. Thus, briquette quality increases with decreasing of feedstock particle size, as indicated in the present study, that the finest particles had greater resistance. However, this trend is limited, according to Kaliyan and Morey [42]. Extremely small particles exhibit a negative influence and decreasing mechanical hardness, which is the main indicator of mechanical quality of briquettes and disproportionately large particles.

On the other hand, the moisture content has a remarkable effect on the briquettes hardness. Previous studies showed that more than 15% moisture content produces poor and weak briquettes [42]. Mani et al. [45] have demonstrated the neediness to establish the initial moisture content of the biomass feed to maintain equilibrium and thus avoid swelling of briquette during storage, transportation, and disintegration when exposed to humid atmospheric conditions. Brunerová and Brožek [46] proved that the moisture of over a long period of stored briquettes changes, namely in dependence on the storage conditions. Feed moisture of 10–12% produces briquettes of 8–10% moisture, and such briquettes are strong and free of cracks [47]. From the above mentioned information, the majority of authors have experiences about briquetting of materials of moisture between 9 and 18%. However, it emerges from the present work that, the moisture content of the base material can drop to around 7%, and still produce briquettes of good quality, with a high capacity to resist shock during transport and storage, but, an excessive drop in the moisture content of the base material can lead to a decrease in the mechanical properties of the briquettes.

The moisture content can vary depending on the particle size of the materials. In this way, Mani et al. [45] and Gilbert et al. [48] proposed a size reduction of raw biomass to obtain better properties of the product by drying, mixing, and briquetting. Biomass material of 6–8 mm size with 10–20% powdery component gives the best results [42]. Mitchual et al. [11] analyzed briquettes produced from tropical hard-wood sawdust, and found that best particle size ranges between 1–2 mm. However, other authors exhibited that suitable particle size ranges between 10–15 mm for briquettes from municipal solid waste [49] or ranges between 6–8 mm size for briquettes made from combination of three hard-wood species [50]. For their part, Tumuluru et al. [51] studied briquettes produced from wheat, oat, canola, and barley straw, indicating the best particle size between 25–32 mm. In the present work on the pecan nut pericarp, the appropriate particle size for the production of good quality briquettes is around 1 mm. Choice of optimal particle size apparently partially depends on concrete feedstock material, but in general, it is not disputed that overall optimal particle size is not defined yet.

## 4. Materials and Methods

### 4.1. Base Materials from Pecan Pericarp Residues and the Biofuel Briquettes Elaboration

The pecan pericarp residues were collected from orchards in southern Nuevo Leon, Mexico, immediately after the harvesting, and were left to dry at room temperature for a minimum time of 10 days. Then, the material was ground through a knife mill and since particles produced by knife milling are not uniform in their size, the particle size distribution was determined according to the UNE-EN 17827-2 standard [52], with a series of metal mesh sieves of standard specifications and sizes, placed in sequential order (from largest to smallest) and arranged from the top to the bottom, to allow a complete reduction of the particle size (12, 20, 40 and 60 corresponding to 1.60 mm, 0.84 mm, 0.42 mm, and 0.25 mm openings, respectively). The representative fraction of each size was collected, and used as raw material for the production of biofuel briquettes. A vertical orientation laboratory briquetting machine (LIPPEL^®^, Agrolândia, Santa Catarina, Brazil) was used, which presents a solid base with a cylinder of 20 mm in diameter and an integrated thermostat that allows the temperature to be modified, two pistons that apply pressure and facilitate the briquette extraction. Each one was made with 200 cm^3^ of the base material, and the operating conditions were 15 MPa of pressure, 90 °C of temperatures and five minutes pressure, similar to that developed by Zepeda-Cepeda [53] and Ramírez-Ramírez [54], for briquettes of 3.3 cm in diameter and 8.0 cm in length. Eight briquettes were made per treatment (granulometry) for subsequent analyzes.

### 4.2. Proximate Analysis and Energy Content of the Pecan pericarp Base Materials

The term “proximate analysis” is used to indicate the ASTM standardized test to define the quality of a fuel [36,55], based on determining the moisture content, volatile matter, ash content, and fixed carbon. Regarding the energy content, the calorific value was determined from the content of fixed carbon (FC) and volatile matter (VM), according to the formula described by Cordero et al. [56]. Although all these properties are somewhat interrelated, they are measured and valued separately, as indicated in Table 1.

### 4.3. Fourier Transform Infrared (FTIR) Spectroscopy for Analysis of Functional Groups from Pecan Pericarp Residues

The phytochemical characterization of the particles was carried out by infrared analysis, using a IFS 66 FT-IR spectrophotometer (Perkin-Elmer Frontier, Waltham, USA) with digitization of spectra that allows to obtain electronic files of the analyzes. All samples were analyzed under the same conditions, using 64 scans, in a range from 4000–400 cm^−1^, at a resolution of 4 cm^−1^. The analysis was carried out with 1 mg of each particle size sample, and the majority functional groups were identified according to the Spectrometric Identification of Organic Compounds manual, and the absorbance of their bands was obtained from a local baseline corrected and normalized at 2900 cm^−1^, between adjacent valleys [57]. Subsequently, the intensities of the spectrometric signals were materialized by calculating the highest point using the OriginLab 8.0 software (OriginLab Corporation, Northampton, MA, USA), with the aim of comparing the availability of possible biofuel compounds in the particles of interest.

### 4.4. Morphological Analysis of Different Particle Sizes from the Pecan Pericarp Residues

The different particle sizes of the pecan pericarp residues materials were analyzed through scanning electron microscopy (SEM). The samples were dehydrated to constant weight, at a temperature of 100 ± 3 °C and to each one, a coating with copper was applied so as not to decompose organic matter, in a Sputter Coater equipment, during a time of 15 min at 10 mAh, to obtain SEM images in a model JSM6400 scanning electron microscope (JEOL, JSM-6400 Tokyo, Japan), coupled to an X-ray spectrometer, with an electron beam acceleration potential of 20 kV at 9.

### 4.5. Physical Properties Analysis of the Biofuel Briquettes from Different Particle Sizes of Pecan Pericarp Residues

#### 4.5.1. Density

The density was determined according to the UNE-EN-16127 standard [58]. Equation (1) was used:

(1) $$\varphi =\frac{m}{V}$$

where φ is the density of the briquettes, m the mass of the sample, and v the volume of the sample.

The volume of the samples was determined by Equation (2):

(2) $$V=\pi r^{2}h$$

Measurements were made after seven days of conditioning at 20 °C and 65% relative humidity, so that the briquette stabilized and that the dilation effect did not affect the results [59].

#### 4.5.2. Hardness

The hardness consisted of estimating and analyzing the briquette ability to resist during the storage, transport and/or compression processes. It is based on weighing each sample, dropping it three times from a height of 1.85 m, and weighing again the piece that remained larger, the calculation of this property was made as described by Kaliyan and Morey [42] with Equation (3):

(3) $$\mathrm{Hardness}=100-\frac{W_{s}-W_{p}}{W_{s}}\times 100$$

where *Ws* is the weight of the briquette sample, *Wp* is the weight of the piece that remained larger.

#### 4.5.3. Swelling

The swelling index was used to see the ability of agglomeration between the particles during the briquetting process. It was determined by Equation (4):

(4) $$\mathrm{Swelling}=\frac{W_{s}-W_{p}}{W_{s}}\times 100$$

where *Ws* is the weight of the briquette sample, *Wp* is the weight of the piece that remained larger.

#### 4.5.4. Impact Resistance Index (IRI)

The impact resistance test simulates the forces encountered during emptying of densified products from trucks onto ground, or from chutes into bins. The IRI was calculated as described by Richards [60] with Equation (5):

(5) $$\mathrm{IRI}=\left(100*N\right)/n$$

where *N* is the number of drops, and n is the total number of pieces after *N* drops. Small pieces weighing less than 5% of the original weight of the logs were not included in the IRI calculation.

### 4.6. Statistical Analysis

Since the data resulting from the proximate analysis are percentage values, they were transformed with the square root function of the *p* arcsine, where *p* = the proportion of the dependent variable [61]. Subsequently, data normality tests were performed for each variable, using the Kolmogorov–Smirnov test. For all the results, a completely randomized experimental design was applied, evaluating 4 treatments with 7 repetitions: T1. N° 12 (1.60 mm), T2. N° 20 (0.841 mm), T3. N° 40 (0.420 mm), T4. N° 60 (0.250 mm), and the statistical package used for the analysis of the data obtained was Origin 2021b. An analysis of variance was performed to verify the significant differences between the variables evaluated, with a 95% confidence interval.

## 5. Conclusions

The proximate analyses carried out on the materials of different particle sizes obtained from pecan (*Carya illinoinensis*) pericarp allowed us to determine the moisture content, volatile matter, ash content, and fixed carbon, which turned out to be adequate to define these materials as good quality biofuels. Its physical transformation through briquetting significantly increased its bioenergetic potential, with calorific values going from 17.00 MJ/kg for the base material to 18.00 MJ/kg for briquettes, due to the high density reached, its hardness and strong impact resistance, giving a solid biofuel that is easy to transport, store, and handle. In this way, the particle size was illustrated as a determining factor in the quality of the briquettes, since the finer materials presented higher density (1.25 g/cm^3^ for granulometry 0.42 mm from a number 40 sieve), and higher hardness (99.85 for biofuel briquettes made from number 60 sieve particle size materials) than larger materials (obtained using number 12 and 20 sieves). Likewise, a greater bioavailability of the main functional groups was registered in the finest materials, which increases the briquettes’ energy characteristics. Based on the results obtained, the studied biomass can produce suitable densified biofuels.

## Figures and Tables

**Table 1:** Parameters analyzed according to the ASTM standards.

	Relation	Observation
Moisture content	$\mathrm{MC}=\frac{\left(\mathrm{Wi}\;–\;\mathrm{Wd}\right)}{\mathrm{Wi}}*100$	Wi = initial weight of the raw material; Wd = dry weight of the raw material after 3 h in an oven at 105 °C.
Volatile matter	$\mathrm{VM}=\frac{\left(\mathrm{Wd}\;–\;\mathrm{Wv}\right)}{\mathrm{Wd}}*100$	Wv = weight of the materials after placing them in a muffle furnace at 950 °C.
Ash content	$\mathrm{Ash}=\frac{\mathrm{Wa}}{\mathrm{Wv}}*100$	Wa = ash weight after muffling at 750 °C during 6 h.
Fixed carbon content	$\mathrm{FC}=100-\left(\mathrm{MC}+\mathrm{VM}+\mathrm{Ash}\right)$	The fixed carbon content was obtained, subtracting the moisture, volatiles, and ash content, from 100%.
Calorific value	CV = 354.3 FC + 170.8 VM	The calorific value is a function of the fixed carbon and volatiles.

## Data Availability

Not applicable.
